# Investigation of the Electrokinetic Potential of Granules and Optimization of the Pelletization Method Using the Quality by Design Approach

**DOI:** 10.3390/pharmaceutics16070848

**Published:** 2024-06-22

**Authors:** Azza A. K. Mahmoud, Alharith A. A. Hassan, Dorina Gabriella Dobó, Krisztina Ludasi, László Janovák, Géza Regdon, Ildikó Csóka, Katalin Kristó

**Affiliations:** 1 Institute of Pharmaceutical Technology and Regulatory Affairs, University of Szeged, Eötvös u. 6., H-6720 Szeged, Hungary; khalid.azza.asim@o365.u-szeged.hu (A.A.K.M.); alharith.hassan@szte.hu (A.A.A.H.); dobo.dorina.gabriella@szte.hu (D.G.D.); ludasi.krisztina@szte.hu (K.L.); geza.regdon@pharm.u-szeged.hu (G.R.J.); csoka.ildiko@szte.hu (I.C.); 2 Department of Physical Chemistry and Materials Science, University of Szeged, Rerrich B. sq. 1, H-6720 Szeged, Hungary; janovakl@chem.u-szeged.hu

**Keywords:** direct pelletization, risk assessment, process validation, electrokinetic properties

## Abstract

The preparation of pellets using a high-shear granulator in a rapid single-step is considered a good economic alternative to the extrusion spheronization process. As process parameters and material attributes greatly affect pellet qualities, successful process optimization plays a vital role in producing pellet dosage forms with the required critical quality attributes. This study was aimed at the development and optimization of the pelletization technique with the Pro-CepT granulator. According to the quality by design (QbD) and screening design results, chopper speed, the volume of the granulating liquid, binder amount, and impeller speed were selected as the highest risk variables for a two-level full factorial design and central composite design, which were applied to the formula of microcrystalline cellulose, mannitol, and with a binding aqueous polyvinylpyrrolidone solution. The design space was estimated based on physical response results, including the total yield of the required size, hardness, and aspect ratio. The optimized point was tested with two different types of active ingredients. Amlodipine and hydrochlorothiazide were selected as model drugs and were loaded into an optimized formulation. The kinetics of the release of the active agent was examined and found that the results show a correlation with the electrokinetic potential because amlodipine besylate can be adsorbed on the surface of the MCC, while hydrochlorothiazide less so; therefore, in this case, the release of the active agent increases. The research results revealed no significant differences between plain and model drug pellets, except for hydrochlorothiazide yield percent, in addition to acceptable content uniformity and dissolution enhancement.

## 1. Introduction

Pellets can be defined as free-flowing, spherical, or semi-spherical particles with a controllable narrow size distribution of an average size of 0.5 mm to 2 mm, intended generally for oral administration. They have advantageous characteristics over single delivery systems, which include free-flowing spherical particles with a smooth texture, optimum drug content, and different drug control-release profiles, in addition to mixing incompatible active pharmaceutical ingredients in the same dosage form. They also improve drug absorption from the GIT and bioavailability with low drug toxicity and low dose-dumping irritation [1,2,3]. Although extrusion spheronization is the most applicable pelletization technique, especially for high drug loading capacity, it is a time-consuming multistep process that requires a high level of control of all process variables during mixing, granulation, extrusion, and spheronization, while the formation of spherical particles by adding a moisturizing liquid to the powder ingredient before or during agitation with a high-shear granulator (direct pelletization) in a single step means less time, cost, and binder liquid consumption with uniform distribution [1,2,3].

The impeller speed is the most important factor that influences the wet granulation technique [4]. It has an effect on the different physical properties of the resulting granules. Many authors reported that increasing impeller speed at a low range value led to a decrease in porosity, size, and friability; moreover, it increased strength and roundness [2]. On the other hand, at a high range value, it produced high-density rigid granules with less roundness and particle size due to the excessive breaking of the granules [2,5,6,7]. In addition, it had an effect on the stability and coating potential of some drugs due to a proportional relationship with process temperature, hardness, and surface free energy [8,9,10]. However, the effect of the impeller was related to the physical properties of the material used, especially binder viscosity because impeller speed affected the distribution of the binder [11], while some authors found that a highly viscous binder produced a small granule size with low porosity and dissolution due to the formation of bridges and kinetic energy between the particles [12]. Others found that granule size increased with a highly viscous binder, leading to reduced consolidation and deformation [6]. On the other hand, it was established that increasing the binder amount resulted in enhancements in granule growth, size, hardness, and flow properties [2] but neither the binder flow rate nor the method of binder addition had an effect on size [5] or granule strength [13]. In contrast, other authors mentioned the different effects of binder flow rate on the size of granules (positive quadratic effect, negative effect) [7,14]. Although the use of a lower impeller speed without a chopper resulted in a large granular size [2], the chopper speed has a significant effect on median particle sizes at high impeller speeds, and it was found that the appropriate selection of a combination of impeller speed and chopper speed contributes to the control of granular size [8,15]. Moreover, using a chopper enhances homogeneity, reduces the size of granules, and subsequently produces a narrow particle distribution [2,11,13] but it has no effect on hardness [10], porosity [13], and process temperatures [8,10].

Even though the water amount had the most significant effect on median diameter (D50), liquid pore saturation, and size, it did not affect the density of granules. Also, using a high water quantity with high impeller speed and long process time produced granules with a narrow size distribution and a large size [16].

The application of quality by design (QbD) to identify and adjust the relationship between critical process parameters (CPPs), critical material attributes (CMAs), and critical quality attributes (CQAs) is considered an essential tool to determine high-risk factors in the process using risk assessment methods such as the Ishikawa diagram. This identifies all sources of risk by determining the cause and effect (qualitative method) and failure mode and effect analysis (FMEA), which estimate the risk value using a combination of severity, probability, and detection (quantitative method) [17,18]. Then, using factorial design to optimize the process, is considered a very important tool to obtain a lot of information about the main factors and their interactions; this has significantly minimized the number of experiments, cost, and time required for the production of a pharmaceutical dosage form with acceptable characteristics—besides the precise determination of design space—which joins all the process parameters and material attribute ranges that ensure obtaining of predetermined critical quality attributes [19,20].

The success of the direct pelletization process can be achieved by combining the QbD approach and design of experiment (DoE) for the precise harmonized selection and control of the process variables that influence the characteristics of the pellets produced with the Pro-CepT granulator, as reported in a few papers [9,10,15].

This study aimed to estimate the effect of direct pelletization process variables on pellet quality and to determine and optimize the design space for low-load drug pellets by using a Pro-CepT granulator. Another aim was to investigate the dissolution and electrokinetic potential of the pellets at the optimized point, as well as their correlations. In our studies, two different types of active ingredients were chosen, one anionic and one cationic, in order to study the behavior, dissolution, and zeta potential of active ingredients with different properties.

## 2. Materials and Methods

### 2.1. Chemicals

In this experimental research, amlodipine besylate and hydrochlorothiazide (Sigma-Aldrich (St.louis, MO, USA)) were chosen as model drugs. Microcrystalline cellulose (MCC, Vivapur 102) was purchased from JRS Pharma (Patterson, NY, USA), while mannitol (Mannitum) and polyvinyl pyrrolidone were supplied by Hungaropharma Zrt. (Budapest, Hungary). Distilled water was used as the granulating binder.

### 2.2. Risk Assessment

QTPPs, CQAs, CMAs, and CPPs were determined based on the previous literature background and preformulation studies, and, then, the risk assessment study was applied using two different methods. The first one was the Ishikawa diagram, which showed all the potential factors that may affect product quality, while the risk estimation matrix (REM) and Pareto chart were made by LeanQbD software (QbD Works LLC, Fremont, CA, USA/Version 1.3.6., 2014) that were used to determine the most important parameters to be considered during the experimental design.

### 2.3. Preparation of Blank Pellets

The pellets were prepared by direct pelletization methods. A total of 60 g of microcrystalline cellulose (MCC), 40 g of mannitol, and different amounts of polyvinylpyrrolidone (PVP) according to DoE were homogenized in a Turbula mixer (Willy A. Bachofen Maschienenfabrik, Basel, Switzerland) for 5 min. The mixture was wetted and kneaded in a Pro-CepT granulator (ProCept NV, ZelZate, Belgium) with water used as a granulation solution. The resulting pellets were dried in an air-ventilated oven (Memmert GmbH+Co. KG, Büchenbach, Germany) at 40 °C for 2 h. According to the DoE results, the design space was determined and optimized using three different parameters within the target values. The optimized formulation was loaded with two APIs (amlodipine besylate and hydrochlorothiazide).

### 2.4. Design of Experiment (DoE)

Design of experiment is a very useful tool to study the effect of process parameters on pellet quality. The studied factors and their levels were shown in Table 1.Three different types of design were carried out, as described below.

#### 2.4.1. Screening Design

A screening design with 8 experiments, 5 factors, and 1 block was carried out using Statistica software, version 13 (TIBCO Software Inc., PaloAlto, CA, USA) (Table 2). Based on the risk assessment results and the previous literature review, 5 parameters were selected as the highest risk factors (impeller speed, chopper speed, granulating liquid amount, binder concentration, and liquid addition rate). The high and low levels of each factor were determined based on preformulation studies, the risk assessment study, and technical aspects.

**Table 1:** Process parameters with selected high, center, and low levels.

Code	Parameter	Low Level (−1)	Center Level (0)	High Level (+1)
**X1**	Impeller speed (rpm)	500	1000	1500
**X2**	Chopper speed (rpm)	1000	1500	2000
**X3**	Liquid volume (mL)	55	57.5	60
**X4**	Binder amount (g)	1	2	3
**X5**	Addition rate (mL/min)	5	7.5	10

#### 2.4.2. Two-Level Full Factorial Design

The 2^4^ factorial designs, with one central point, were applied based on Statistica (Table 3). Impeller speed (X_1_), chopper speed (X_2_), granulating liquid amount (X_3_), and binder amount (X_4_) were used as dependent variables, while aspect ratio, size yield percent, hardness, and friability were used as independent variables.

#### 2.4.3. Central Composite Design

An additional 10 experiments were performed according to the central composite design (Table 4) using the same dependent and independent variables as previously mentioned in the two-level full factorial design.

### 2.5. Physical Characteristics of Pellets

#### 2.5.1. Size Distribution and Yield Percent

The pellets of each sample were sieved with a series of sieves (Retsch GmbH, Haan, Germany) in the range of between 500 µm and 1400 µm for 20 min. The amount of sample in each sieve was weighed and the total sample percentage amount between 710 µm and 1120 µm was recorded. The yield percent was calculated according to the equation:

Yield percent = (final pellet weight/initial powder weight) × 100

#### 2.5.2. Aspect Ratio and Roundness

Aspect ratio and roundness were measured for 10 pellets using a stereomicroscope (Carl Zeiss, Oberkochen, Germany) and Leica Quantimet 500 C image analysis software (Leica Microsystems, Wetzlar, Germany) according to the following equations:

Aspect ratio = D_max_/D_min_

Roundness = Perimeter^2^/4π × Area

#### 2.5.3. Hardness and Deformation Properties

The breaking force and deformation behavior were determined for ten pellets of each sample within the size range of 710–1120 µm using a texture analyzer with a measuring force between 0 and 200 N and a probe speed of 20 mm/min. The mean breaking force was calculated.

### 2.6. Drug-Loaded Pellet Test

#### 2.6.1. Dissolution Test

The dissolution test of amlodipine besylate and hydrochlorothiazide was performed using a dissolution apparatus (Ereweka DT 700, Heusenstamm, Germany) in 900 mL of different media (HCL (0.1N) and phosphate buffer (pH 6.8)) at 37 ± 0.5 °C and 100 rpm as the rotation speed. Three milliliters were removed from each flask and replaced with the same volume of fresh medium at different time intervals (5, 10, 15, 20, 30, 45, and 60 min) for amlodipine besylate pellets and (5, 10, 15, 20, 30, 45, 60, 75, 90, and 100 min) for hydrochlorothiazide pellets. The absorbance of amlodipine besylate and hydrochlorothiazide pellets was recorded at 238 nm and 272 nm, respectively, with a UV–Vis spectrophotometer (Unicam Helios α, Spectronic Unicam, Budapest, Hungary). Then, the cumulative drug release percent was calculated using calibration curve equations (Figures S5 and S6).

#### 2.6.2. Electrokinetic Potential Measurement (SurPass)

The surface electrokinetic zeta potential of the granule and powder samples was determined with a SurPASS 3 Instrument (Anton Paar, Graz, Austria) in PBS buffer and distilled water. The samples were placed in a powder cell with an adjustable part in contact with the continuously flowing liquid medium. The measurement was performed at 20 consecutive points, at which point the zeta potential value stabilized. The last value was taken into account.

#### 2.6.3. Content Uniformity

Pellets with a weight equivalent to 5 mg of pure amlodipine besylate and 25 mg of hydrochlorothiazide were placed in a volumetric flask and ultrasonicated for 10 min. The volume was completed with 10 mL methanol for amlodipine pellets and 100 mL NaOH (0.01N) for hydrochlorothiazide pellets, and both solutions were filtered. Then 1 mL of each solution was diluted 10-fold. The absorbance of amlodipine besylate and hydrochlorothiazide samples was recorded at λ max 238 nm and 272 nm, respectively, and the drug content was calculated. Calibration curves of amlodipine besylate and hydrochlorothiazide were prepared using serial concentration 3, 5, 7, 9, 11 µg/ml for amlodipine besylate and 5, 10, 15, 20, 30 µg/ml for hydrochlorothiazide (Figure S4).

#### 2.6.4. FT-IR Spectroscopy

A small amount of each sample (amlodipine besylate powder, hydrochlorothiazide powder, amlodipine pellets, hydrochlorothiazide pellets, microcrystalline cellulose, mannitol, and polyvinyl pyrrolidone) was mixed with 0.2 g KBr powder, then, it was compressed as a disk and analyzed using FT-IR spectra (Avatar330 FT-IR (Thermo Fisher Scientific Inc., Waltham, MA, USA) at a wavelength of 600–4000 cm^−1^. The spectra were collected from 128 scans to obtain smooth spectra, at the spectral resolution of 4 cm^−1^, and applying CO_2_ and H_2_O corrections.

## 3. Results and Discussion

### 3.1. Risk Assessment

The application of risk assessment during the pelletization process with a high-shear granulator is considered an excellent option that overcomes the huge challenge of controlling a large number of process variables as it has a beneficial effect on understanding each process stage besides the accurate selection of process parameters, materials attributes, and critical quality attributes through different methods (Ishikawa diagram, risk estimation matrix, and Pareto chart) to ensure the preparation of pellet dosage forms of the desired quality.

#### 3.1.1. Ishikawa Diagram

**Figure 1:**
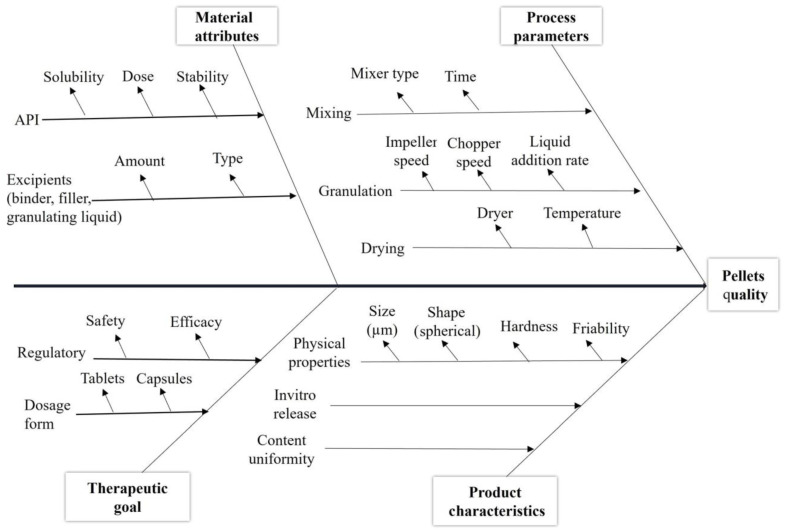
Ishikawa diagram for determining critical factors for direct pelletization by using Pro-CepT granulator (definition and classification of all expected factors).

An Ishikawa diagram (Figure 1) defined and classified all the expected factors that could affect the quality of the resulting pellets into four groups, which included material attributes, process parameters, product characteristics, and the therapeutic goal.

#### 3.1.2. Risk Estimation Matrix and Pareto Chart

**Figure 2:**
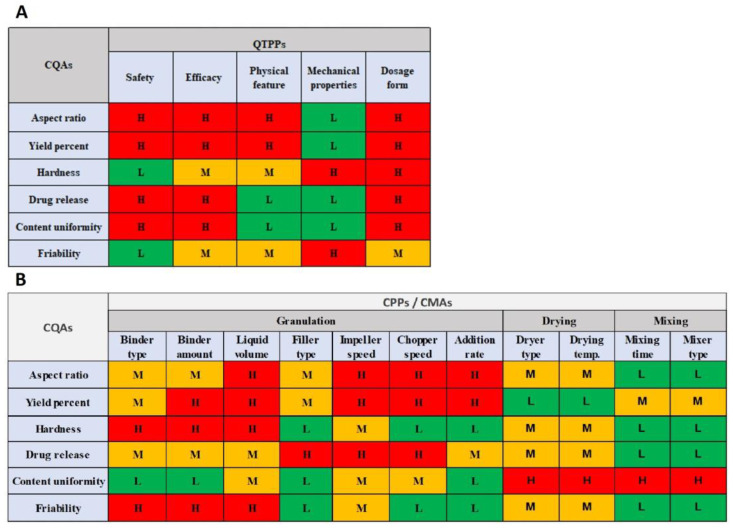
Matrix diagram, (**A**). Effect of quality target profile (QTTP) with critical quality attributes (CQAs), (**B**). Critical quality attributes (CQAs) with critical material attributes (CMAs)/critical process parameters (CPPs) using three grades: red color—high (H), yellow color—medium (M), and green color—low (L) for direct pelletization techniques.

After all the process variables were estimated in the Ishikawa diagram, the second step was to identify the QTPPs and CQAs (Table 5 and Table 6) and then to explain the relationship between them through evaluating the effect of critical process parameters (CPPs)/critical material attributes (CMAs) on critical quality attributes (CQAs), besides the relationship between the quality target product profile (QTPP) and critical quality attributes (CQAs). This was carried out using a risk estimation matrix (REM) (Figure 2) in three grades: high (H), medium (M), and low (L). These grades were determined based on preformulation studies, group experiments, and studies in the literature.

**Table 5:** Quality target product profile (QTPP) of direct pelletization with ProCepT granulator.

QTPP	Goal	Justification
**Morphological feature**	Spherical particles with a narrow size distribution	To enhance flow properties during the manufacturing process, solubility, and release kinetics of the drug [21,22,23]
**Mechanical properties**	High tensile strength Low friability	Pellets must be able to withstand mechanical forces during various technological processes, such as filling and coating [9]
**Efficacy**	Optimum content uniformity	Multiparticulate particles are freely distributed in the gastrointestinal tract, which leads to enhanced absorption, in addition to the control of drug release according to the required purpose [1,21,22].
**Safety**	Low toxicity	Small particle size reduces dose dumping and, consequently, gastrointestinal tract irritation and drug toxicity [23,24,25]
**Dosage form**	Capsule and tablet	Most applicable dosage forms due to accuracy, stability, and patient compliance [26]

**Table 6:** Critical quality attributes (CQA) of pellets prepared by direct pelletization.

CQA	Goal	Justification
**Size**	500–1500 µm	To minimize segregation hazard and better coating [21,24]
**Aspect ratio and roundness**	<1.2	Spherical particles have good flow properties, which is a critical factor in the preparation of solid dosage forms [27]
**Hardness**	Within a good range	To ensure good tableting compression, capsule-filling, and coating [27,28]
**Friability**		
**Angle of repose**	25–40	Good rheological properties can be used as an indicator of the degree of sphericity. On the other hand, flow properties with compressibility behavior are very important for the direct compression of tablets and filling of capsules [21,23]
**Hausner factor**	1–1.34	
**Cumulative drug release percent**	>80%	To ensure high drug absorption and bioavailability [29]
**Content uniformity**	Within the required range (depending on the drug)	To obtain an optimum therapeutic effect with the lowest toxicity and side effects

**Figure 3:**
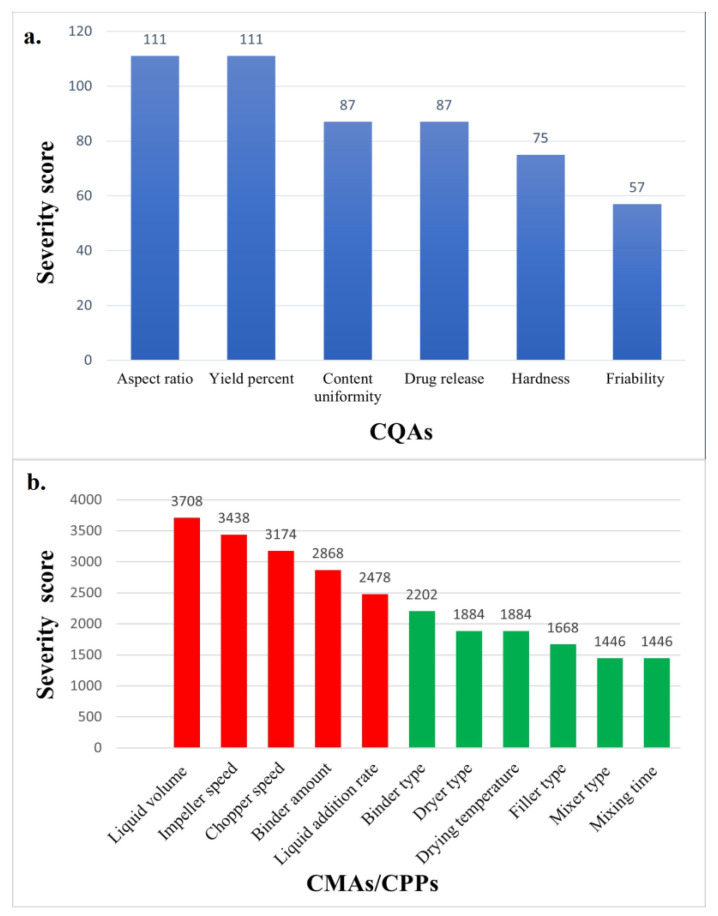
Pareto chart effect, (**a**). Quality target profile (QTTP) with critical quality attributes (CQAs), (**b**). Critical quality attributes (CQAs) with critical material attributes (CMAs)/critical process parameters (CPPs) using two grades: red color—high (H), and green color—low (L) for direct pelletization techniques.

According to the risk assessment results based on the risk estimation matrix and the Pareto chart (Figure 2 and Figure 3), impeller speed, chopper speed, granulating liquid amount, liquid addition rate, and binder concentration were selected as independent variables for further studies by DoE, while the dependent variables of the experimental design were size distribution, aspect ratio, and hardness due to their highest severity values.

### 3.2. Preparation of Blank Pellets

The type of excipients plays a very important role in the pelletization technique based on their physicochemical properties that could affect the quality of formulated pellets. According to the previous literature and preformulation studies, it was found that the use of microcrystalline cellulose (MCC) as a filler is beneficial in manufacturing pellet dosage forms due to its excellent properties of high water uptake, cohesive behavior, plastic texture, and disintegration into small particles; this results in a more spherical and smoother pellet surface than other fillers due to the gel formation before the drying of the pellets [16]. However, MCC has the drawback of elongation of the dissolution time of poorly soluble drugs such as hydrochlorothiazide, and this could be overcome by PVP, which has high disintegration properties and subsequently improves dissolution besides minimizing water quantity during the manufacturing process due to its good binding effects. Generally, binders are essential excipients in pelletization processes, especially in particle consolidation and maintaining a uniform drug distribution. Furthermore, the use of mannitol as a secondary filler, which also has high water solubility, improves flow properties, minimizes bulk density, and contributes to enhancing drug dissolution [30,31].

### 3.3. Design of the Experiment

ANOVA was used to examine the significance of the quadratic model on the responses (linear, interactive, and polynomial). The *p* values for each of the five responses were listed along with the impact of the model variables (R^2^, adjusted R^2^, and MS residual). A model was considered significant at the 95% confidence level if the *p* value was less than 0.05. The sign and value show the magnitude of the influence on the response. Negative signs indicate a decline in the response value, while positive signs show an increase in it.

#### 3.3.1. Screening Design

The primary benefit of screening designs is the reduction in the number of variables by excluding those that have little or no impact on the CQAs and maximizing the information collected from the process. The relationship between the process factors and responses, which include aspect ratio (Y_1_), yield percent (Y_2_), and hardness (Y_3_), are described in Equations (1)–(3) (*p* < 0.05).

Y_1_ = **1.626** + **0.03025X_1_** + **0.06325X_2_** + **0.21375X_3_** + **0.0465X_4_** − 0.0025X_5_ − **0.0765X_2_X_3_** (1)

R^2^ = 0.99999, Adjusted R^2^ = 0.99993, MS Residual = 0.0000045

Y_2_ = **39.25 − 3.25X_2_ − 22.25X_3_ + 4.75X_4_** + 2.25X_2_X_3_ − 1.25X_2_X_4_ (2)

R^2^ = 0.9979, Adjusted R^2^ = 0.99265, MS Residual = 4.5

Y_3_ = **35.76963** − **0.57513X_1_** − **0.88987X_2_** − 0.43938X_3_ + **3.01137X_4_** − 0.18512X_5_ − **1.29713X_2_X_4_** (3)

R^2^ = 0.99989, Adjusted R^2^ = 0.99922, MS Residual = 0.0107311

According to the screening design results (Table 7), chopper speed and binder amount were the most important factors and had a significant effect on all variables due to their effect on clumps breaking, size reduction below the required size, and enhancement of binder distribution, which subsequently led to the enhancement of hardness, particle growth mechanism, and flow properties [18]. Also, impeller speed and the volume of the granulating liquid had a significant effect on some variables. This effect was apparent in the positive effect of binder amount on hardness and yield percent independently of other factors, in addition to the experiments containing 55 mL of liquid volume (the low level) that showed a lower aspect ratio and higher yield percent with no significant effect on hardness. Moreover, it was noticed that the effect of chopper speed is greatly related to water volume and increasing chopper speed decreases the hardness and aspect ratio and increases yield percent at low water volume (55 mL) as it minimizes particle size within the required range; however, it had an opposite effect at higher water volumes due to the aggregation of particles and subsequent loss of spherical shape and increase in pellet size above the required size. In spite of the negative effect of the addition rate of the granulating liquid on the aspect ratio and hardness (increase in size and porosity), it had no significant effect on any of the dependent variables, and this result is in agreement with the previous literature [32]. Therefore, it was fixed at a low level (5 mL/minute) and the independent variables were reduced to four factors, which were impeller speed, chopper speed, granulating liquid volume, and binder amount in the next experimental design step.

#### 3.3.2. Two-Level Full Factorial Design

As the screening design results revealed the intervening effect of process variables on pellet properties, the application of a two-level full factorial design was primitive to accurately determine the effect of each factor and the interaction between them on the selected responses. Also, the addition of one center point experiment was included to enhance the precision of analysis besides the detection of the possibility of curvature behavior [19]. The two-level full factorial design results (Table 8) were analysed and the best-fit Equations (4)–(6) for aspect ratio (Y_1_), yield percent (Y_2_), and hardness (Y_3_), respectively (*p* < 0.05), were determined based on the highest R^2^, highest adjusted R^2^, and lowest MS residual.

Y_1_ = **1.629625** − **0.409625C** + **0.116750X_1_** + **0.020250X_2_** + **0.217125X_4_** + **0.046125X_1_X_2_** + **0.0055X_1_X_3_** + **0.086X_1_X_4_** + **0.04X_2_X_3_** − **0.0865X_2_X_4_** − **0.13125X_3_X_4_** − **0.049125X_1_X_2_X_3_** + **0.042875X_1_X_2_X_4_** + **0.029X_1_X_3_X_4_** − **0.016X_2_X_3_X_4_** (4)

R^2^ = 0.99998, Adjusted R^2^ = 0.9998, MS Residual = 0.0000223

Y_2_ = **38.125 + 25.875C − 4.625X_1_ + 2.5X_2_ − 15.375X_3_** − **11.375X_4_ − 2.125X_1_X_3_** + 0.375X_1_X_4_ + **1.25**X_2_X_3_ + 2.25X_2_X_4_ + **2.625X_3_X_4_** + 0.5X_1_X_2_X_3_ + **2.25X_1_X_2_X_4_ + 3.125X_1_X_3_X_4_** + **1X_2_X_3_X_4_** (5)

R^2^ = 0.99987, Adjusted R^2^ = 0.99893, MS Residual = 0.5

Y_3_ = **34.11465** − 2.38456C + **0.84919X_1_** + **1.22056X_2_ + 1.95581X_3_** + **1.56156X_4_ − 0.86731X_1_X_2_ + 1.13919X_1_X_4_** − 0.43431X_2_X_3_ + **1.11906X_2_X_4_** + 0.44831X_3_X_4_ + **0.99881X_1_X_2_X_3_** + 0.36319X_1_X_2_X_4_ + 0.66369X_1_X_3_X_4_ − 0.30919X_2_X_3_X_4_ (6)

R^2^ = 0.99541, Adjusted R^2^ = 0.96328, MS Residual = 0.5226571

In accordance with previous studies, hardness is significantly affected by high impeller speed, which ensures the uniform distribution of the binder and granulating liquid volume, and they also have a significant positive effect on pellet hardness because of their vital roles in enhancing cohesive force and increasing particle aggregation [6]. Moreover, chopper speed has a positive significant effect on hardness, which is not in agreement with Kristó et al. and may be related to its effect on reducing the size of the granules. On the other hand, aspect ratio is positively affected by impeller speed, chopper speed, liquid volume, and some two-way and three-way interactions. It was found that decreasing the granulating liquid contributed to the reduction in particle size and clump formation, which has a beneficial effect on reducing the aspect ratio below 1.2 and enhancing roundness; however, a very high impeller speed enhances porosity, particle growth, and leads to less spherical rigid granules with a high aspect ratio [2,16]. Moreover, high chopper speeds cause the breakdown of granules and consequently reduce their roundness. In contrast, the yield percent results show that liquid volume has a negative effect on yield percent, which obviously appeared in runs number 9, 10, 11, and 13 and were carried out using a higher level of granulation liquid volume (60 mL) and lower binder amount (1 g); this resulted in a low yield percent <20% due to over-wetting and clump formation and subsequently enlargement of particles above the required size range [17]. Also, using an insufficient amount of binder might lead to failure in the formation of the bridges between particles that are necessary for the formation of pellets to have the required size and shape. The central point experiment indicated a significant curvature in the aspect ratio and yield percent results, which means a non-linear relationship between the critical process parameter and two critical quality attributes.

#### 3.3.3. Central Composite Design

The extension of the two-level design with a central composite design with minimal extra experiments provided additional information about the non-linear relationship between PPAs and CQAs (Table 9) (Figures S1–S3) [33]. This was revealed by the significant coefficient of linear and quadratic models in the best-fit second-order Equations (7)–(9) of aspect ratio (Y_1_), yield percent (Y_2_), and hardness (Y_3_), respectively (*p* < 0.05).

Y_1_ = 1.224271 + **0.194722X_1_** + **0.353415X_2_^2^** + **0.107556X_3_ + 0.049915X_4_^2^ + 0.131125X_1_X_2_ + 0.086X_1_X_3_ − 0.0865X_1_X_4_** + 0.04X_2_X_4_ + 0.046125X_3_X_4_ (7)

R^2^ = 0.9141, Adjusted R^2^ = 0.8658, MS Residual = 0.0134704

Y_2_ = **63.25 − 14.3333X_1_ − 11.5556X_2_ − 24.8056X_2_^2^** − **5.3333X_3_ − 2.2778X_4_** + 2.625X_1_X_2_ − 2.25X_1_X_4_ (8)

R^2^ = 0.93101, Adjusted R^2^ = 0. 90418, MS Residual = 42.43056

Y_3_ = 32.7443 + **1.4023X_1_** + 0.9498X_1_^2^ + **1.9642X_2_** − 0.9602X_2_^2^ + **0.9361X_3_ + 1.34511X_4_** + 1.4298X_3_^2^ + **1.1137X_1_X_3_ + 1.1445X_1_X_4_** − 0.8927X_3_X_4_ (9)

R^2^ = 0.83498, Adjusted R^2^ = 0.72496, MS Residual = 2.919908

**Figure 4:**
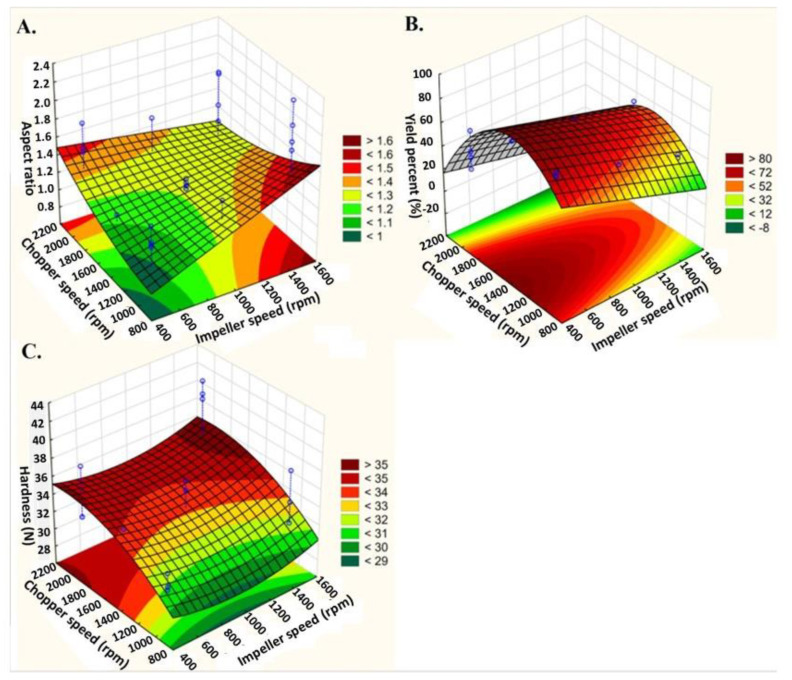
Three-dimensional response surface plots showing the effects of impeller speed and chopper speed at constant liquid volume (55 mL) and binder amount (2 g) on (**A**) aspect ratio, (**B**) yield percent, and (**C**) hardness.

Although the use of a central composite design led to a decrease in coefficient values due to the increasing number of experiments, it advantageously revealed a clearer picture of the linear and quadratic relationship between process variables and responses [20]. It was found that impeller speed (X_1_) and the volume of the granulating liquid (X_3_) have a significant effect on all responses (Table 10). On the other hand, during the application of the central composite design, it was found that only the quadratic (non-linear) chopper speed (X_2_^2^) has a positive effect on the aspect ratio, while the linear chopper speed (X_2_) has no effect on the aspect ratio. In addition, the effects of both factors (linear and quadratic chopper speed) can reduce the yield percentage. Furthermore, it found that only the quadratic binder concentration (X_4_^2^) positively affected the aspect ratio, while the linear binder concentration (X_4_) negatively affected the yield percent, which confirmed and explained the presence of curvature in the two-level design results for these aspects. Also, it was found that the interaction between impeller and chopper speed (X_1_X_2_) has a significant positive effect on aspect ratio, similar to the main effects. Therefore, simultaneous use of high levels of impeller and chopper speeds resulted in pellets of low roundness (a high aspect ratio) (Figure 4).

According to central composite results, there is a significant proportional relationship between linear impeller speed, linear granulating liquid volume, and quadratic chopper speed, with an aspect ratio consistent with the two-level design results. In contrast, yield percent was negatively affected by linear impeller speed, linear granulating liquid volume, linear and quadratic chopper speed, as low chopper speeds produced smaller particles with a narrow particle size distribution, while the use of very high chopper speeds led to the breaking down of granules [8,15]. Also, hardness was significantly affected by linear impeller speed, linear chopper speed, linear liquid volume, and linear binder amount, which confirmed the two-level results of non-significant curvature or the absence of a non-linear relationship for the same reasons as mentioned in the two-level design.

#### 3.3.4. Process Optimization and Validation

**Figure 5:**
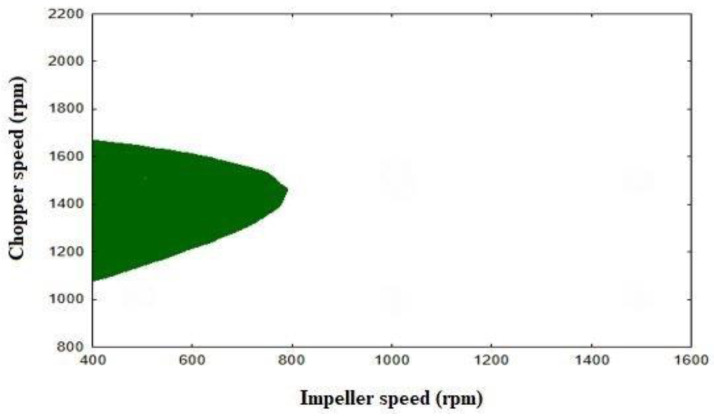
Design space for direct pelletization with Pro-CepT granulator.

A design space was constructed to obtain pellets with required CQAs, which included the following: aspect ratio <1.2, yield percent >80%, and hardness <33 N (Figure 5). Impeller speed and chopper speed were chosen as the main variables (X, Y axis) in the design space because they had higher significant *p* values and effects on the size of the design space than other factors.

Although liquid volume had a positive significant impact on hardness, it was fixed at a low level (55 mL) because it had a negative effect on yield percent and roundness (increasing aspect ratio > 1.2), while binder amount was fixed at center level (2 g), which increased the design space. The last step in the design of experiment was model validation, which was tested at three different experimental points within the design space, and it showed that the design space had good robustness and the results were within the predetermined design space ranges with small variation between the practical and predicted results (Table 11).

### 3.4. Drug-Loaded Pellets

#### 3.4.1. Physical Tests

The optimized experiment was selected from the design space (impeller speed 500 rpm, chopper speed 1500 rpm, binder amount 3 g, and liquid amount 55 mL), loaded with amlodipine besylate and hydrochlorothiazide drugs using the same experimental values and process conditions (Table 12).

**Figure 6:**
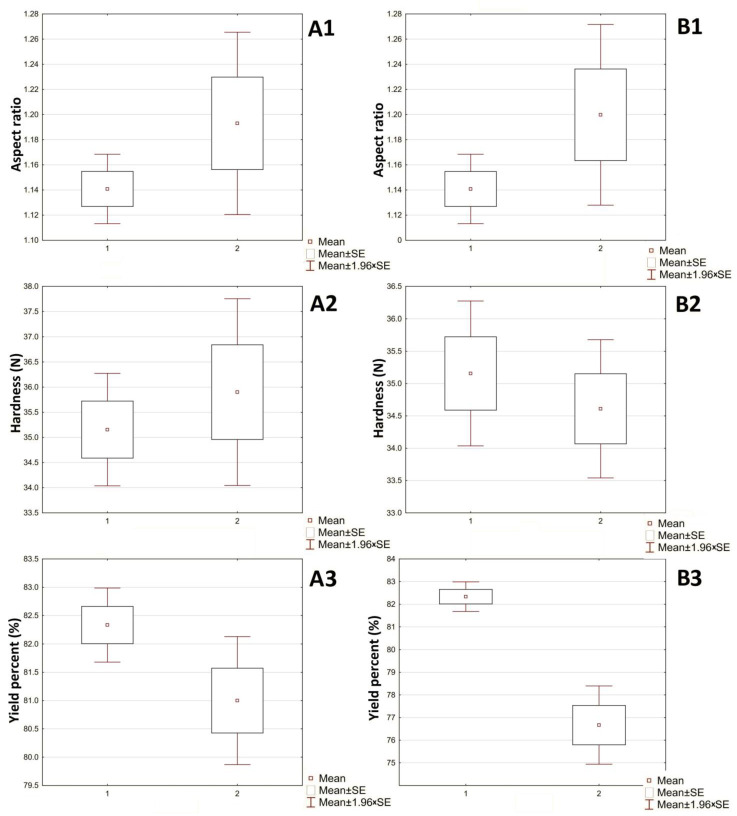
*T*-tests of the physical properties of drug-free pellets: (**A1**–**A3**) Amlodipine besylate pellets; (**B1**–**B3**) Hydrochlorothiazide pellets.

A *t*-test (Figure 6) was carried out to estimate the effects of the model drugs on the critical physical quality attributes of the pellets, which included yield percent, hardness, and aspect ratio (Figure 5). It was revealed that the addition of amlodipine besylate and hydrochlorothiazide did not significantly affect aspect ratio and hardness (*p* value < 0.05); however, it had a significant effect on the yield percent of hydrochlorothiazide due to the higher drug amount of hydrochlorothiazide—which displaced microcrystalline cellulose and mannitol more than amlodipine—in addition to the hydrophobic nature of hydrochlorothiazide that may reduce wettability of the powder mixture by granulating liquid and, consequently, reduce the yield percent [34].

#### 3.4.2. Content Uniformity Results

The mean drug content of amlodipine besylate and hydrochlorothiazide was 98.05 ± 0.2% and 98 ± 0.41%, respectively, indicating uniform drug distribution due to the optimum mixing of the three-dimensional movement of the Turbula mixer, besides the mixing effect of the high-shear granulator.

#### 3.4.3. Dissolution Test

**Figure 7:**
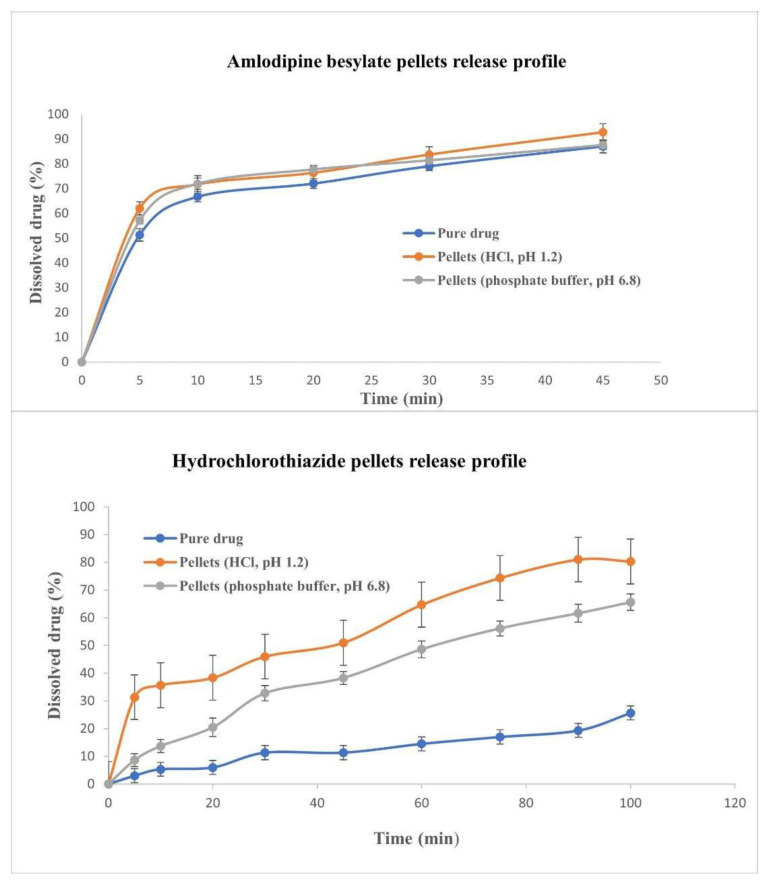
Dissolution release profile for amlodipine besylate pellets and hydrochlorothiazide pellets.

Based on the results of the dissolution profiles (Figure 7), 92.6 ± 1.6% of amlodipine besylate was released within 45 min, while 80.3 ± 2.08% of hydrochlorothiazide was released within 100 min. Pellets had a better dissolution rate than pure drugs; this is related to the beneficial effect of the multiparticle system in improving drug dissolution, in addition to enhancing the wettability of pellets due to the hydrophilic nature of polyvinylpyrrolidone and mannitol, which also form a glass solution with the hydrophobic drug and increase the microporosity of the resulting pellets and, consequently, enhance drug release [5,34]. The kinetic of hydrochlorothiazide based on the above-mentioned models was estimated and best fitted to zero order (R^2^ 0.9824) and Hopfenberg (R^2^ 0.9824) models in acidic medium; however, it changed to a first-order model in phosphate buffer, which may be related to the change in solubility behavior at different pHs of dissolution medium. For amlodipine, the Hixon–Crowell model fitted best in both media (R^2^ 0.9832 and 0.9959).

**Table 12:** Physical test results of amlodipine besylate and hydrochlorothiazide [31].

Test	Amlodipine Besylate	Hydrochlorothiazide	Drug-Free Pellet
**Aspect ratio**	1.14	1.19	1.192
**Yield percent (%)**	82	79	83.36
**Hardness (N)**	35.89	37.86	34.02

The SurPass apparatus enables the direct analysis of the zeta potential at the interface of solids/liquids by means of flow potential or flow current measurements. This, in turn, provides access to the entire range of zeta potential of technical materials down to a few millivolts and surface charge information, with high reliability and repeatability.

**Figure 8:**
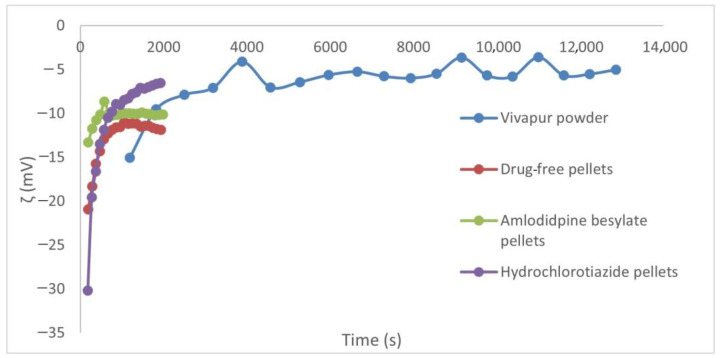
Results of electrokinetic potential measurements (SurPass measurements).

Figure 8 shows that the measurement of the Vivapur powder took much longer than the measurement of the granules. The reason for this is to be found in the particle size because according to the manufacturer’s data, the particle size of Vivapur 100 is around 60 μm, which after compression in the cell creates a compact structure through which the PBS buffer can flow more slowly. Whereas, in the case of pellets, due to the large particle size, a relatively large volume of empty space remains between the pellet particles in the cell, through which the liquid can flow easily and quickly. After contact with the PBS buffer, the pellets slowly start to disintegrate but in this case, the fluid flow does not slow down either because the volume of the cell remains constant; therefore, the flowing PBS buffer can easily and quickly flow through the cell between the disintegrating and the disintegrated loose particles.

When comparing the pellets, it can also be seen that amlodipine besylate slightly changes the zeta potential value of the pellets, while hydrochlorothiazide increases it (Table 13). This is consistent with the dissolution profiles, as Figure 7 shows that while the dissolution curves are almost identical for amlodipine besylate, there is a significant increase in dissolution for hydrochlorothiazide.

Table 13 shows the values measured at the last point of the surpass measurements. During the evaluation, we compared these in the case of Vivapur and pellets without the active ingredient.

Adsorption of active ingredients on solid surfaces can affect dissolution, such as in this case adsorption on the MCC surface. While hydrochlorothiazide has a higher affinity for positively charged micelles [34], amlodipine besylate binds more to the negatively charged cellulose surface [35]. At the same time, it is important to note that not only electrostatic interactions play a role but also, for example, the hydrophilic and lipophilic properties of the materials and other excipients. However, the correlation between the dissolution and the zeta potential results is visible because in the case of hydrochlorothiazide—where it was possible to increase the dissolution—the zeta potential values are more around the value of pure Vivapur, while in the case of amlodipine besylate, the zeta potential of the granules without the active ingredient is closer to its value. In the latter case, some interactions are assumed, even in the case of granules without the active ingredient, with the excipients. In the case of amlodipine besylate pellets, amlodipine also can interact electrostatically with MCC [35], and in this case, it was not possible to increase the release of the active ingredient. Therefore, the dissolution kinetics can also be affected by adsorption on solid surfaces, for which the measurement of the electrokinetic potential can be a promising method.

**Table 13:** Results of SurPass measurements in PBS buffer.

Sample	Zeta potential (mV)
**Vivapur powder**	−5.01
**Drug-free pellets**	−11.87
**Amlodipine besylate pellets**	−10.13
**Hydrochlorothiazide pellets**	−6.55

#### 3.4.4. FT-IR Spectroscopy

**Figure 9:**
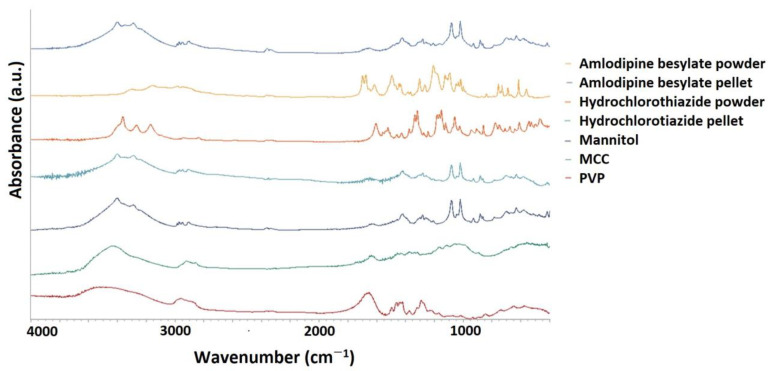
FT-IR spectroscopy of amlodipine besylate pellets, hydrochlorothiazide hydrochloride pellets, and excipients (MCC, PVP, and mannitol).

FT-IR (Figure 9) was used to study the chemical changes and interactions of drugs. For the amlodipine besylate pellet sample, the two peaks displayed medium intensities between 3300 and 3400 cm^−1^ and the single peak between 3200 and 3300 cm^−1^ indicates the presence of the stretching of primary and secondary amines, while the peak appearing at 3400 cm^−1^ is related to the OH groups of mannitol and microcrystalline cellulose. The aliphatic CH_3_ stretching was detected between 2800 and 3000 cm^−1^. The overlapping peak between 1600 and 1800 cm^−1^ resulted from aromatic skeletal stretch, the ester carbonyl group of the drug in addition to the aromatic ketone of the polyvinyl pyrrolidone polymer. On the other hand, the peak above 1400 cm^−1^ can be attributed to CH_3_-CH_2_ bending. The absorption peaks of aromatic C-Cl and asymmetric sulfonate (S=O) stretching appeared at around 1000 and 1207 cm^−1^, respectively [36].

For the hydrochlorothiazide pellet, the same peaks of the hydroxyl group of mannitol and microcrystalline cellulose appeared at 3400 cm^−1^. Also, symmetric aliphatic CH_2_, a doublet primary amine, and a single peak of secondary amines were found between 2900 and 3000 cm^−1^ as well as 3300 and 3200 cm^−1^. Furthermore, the stretching of heterocyclic sulphonate and asymmetric sulphonate appeared between 1000 and 1200 cm^−1^, while CH_3_-CH_2_ bending and a C-Cl peak were located at 1400 cm^−1^ and 850 cm^−1^ [37,38]. All these results suggest that no interaction can be found between the different active ingredients and the excipients. Basically, the characteristic peaks of the excipients are visible, while the peaks of the active ingredient are less visible in the pellets due to the low concentration of the active ingredient (Figure 9).

## 4. Conclusions

In this study, a successful direct pelletization was carried out in a single step using a Pro-Cept granulator. The QbD approach was applied to estimate the highest-risk process variables including binder amount, liquid volume, liquid addition rate, chopper speed, and impeller speed. The screening design also played a vital role in estimating the lower and upper levels of each process variable and excluding the binder addition rate, which had a non-significant effect on all process responses. On the other hand, a two-level full factorial design and a central composite design were applied to optimize the process by determining the design space with required responses of high total yield within optimum size (710–1120 µm) in addition to aspect ratio <1.12 and high hardness <33 N, which was validated for three different points within the design space, and the point with the best formula was loaded with two different model drugs: amlodipine besylate and hydrochlorothiazide. This resulted in approximately the same sphericity and hardness of drug pellets as plain pellets but there was a significant difference in yield percent for hydrochlorothiazide pellets due to the hydrophobic nature of the drug. It was found that in the case of the hydrochlorothiazide pellet, the release of the active ingredient improved, which showed a good correlation with the electrokinetic potential results. Moreover, both drugs had acceptable content uniformity, making them a promising option for formulating low-load drugs as pellet dosage forms, with extensive studies of environmental conditions within granulators and overcoming scaling-up obstacles.

## Figures and Tables

**Table 2:** Screening design.

Experiment No.	Impeller Speed (rpm)	Chopper Speed (rpm)	Liquid Addition Rate (mL/min)	Granulating Liquid Volume (mL)	Binder Amount (g)
**1**	500	1000	10	55	3
**2**	1500	1000	5	55	1
**3**	500	2000	5	55	3
**4**	1500	2000	10	55	1
**5**	500	1000	10	60	1
**6**	1500	1000	5	60	3
**7**	500	2000	5	60	1
**8**	1500	2000	10	60	3

**Table 3:** Two-level full factorial design.

Experiment No.	Impeller Speed (rpm)	Chopper Speed (rpm)	Granulating Liquid Volume (mL)	Binder Amount (g)
**1**	500	1000	55	1
**2**	1500	1000	55	1
**3**	500	2000	55	1
**4**	1500	2000	55	1
**5**	500	1000	55	3
**6**	1500	1000	55	3
**7**	500	2000	55	3
**8**	1500	2000	55	3
**9**	500	1000	60	1
**10**	1500	1000	60	1
**11**	500	2000	60	1
**12**	1500	2000	60	1
**13**	500	1000	60	3
**14**	1500	1000	60	3
**15**	500	2000	60	3
**16**	1500	2000	60	3
**17(c)**	1000	1500	57.5	2

**Table 4:** Central composite design.

Experiment No.	Impeller Speed (rpm)	Chopper Speed (rpm)	Granulating Liquid Volume (mL)	Binder Amount (g)
**18**	1500	1500	57.5	2
**19**	1000	1000	57.5	2
**20**	1000	2000	57.5	2
**21**	1000	1500	55.0	2
**22**	1000	1500	60.0	2
**23**	1000	1500	57.5	1
**24**	1000	1500	57.5	3
**25**	1000	1500	57.5	2
**26**	1000	1500	57.5	2
**27**	500	1500	57.5	2

**Table 7:** Screening design results.

Experiment No.	Aspect Ratio	Yield Percent (%)	Hardness (N)
**1**	1.287	73	41.834
**2**	1.258	61	32.437
**3**	1.57	58	37.757
**4**	1.534	54	32.808
**5**	1.773	12	32.265
**6**	1.933	24	40.102
**7**	1.753	11	33.523
**8**	1.9	21	35.431

**Table 8:** Two-level full factorial design results.

Experiment No.	Aspect Ratio	Yield Percent (%)	Hardness (N)
**1**	1.204	69	30.938
**2**	1.258	61	32.437
**3**	1.229	61	30.593
**4**	1.436	71	30.214
**5**	1.524	67	32.084
**6**	1.237	78	34.347
**7**	1.57	58	37.757
**8**	1.842	35	32.054
**9**	1.845	11	30.559
**10**	2.292	13	31.556
**11**	1.753	11	33.523
**12**	2.023	27	37.45
**13**	1.582	7	31.129
**14**	1.933	24	40.102
**15**	1.396	54	39.54
**16**	1.95	20	41.55
**17 (c)**	1.22	65	31.73

**Table 9:** Central composite results.

Experiment No.	Aspect Ratio	Yield Percent (%)	Hardness (N)
**17**	1.184	73	33.96
**18**	1.215	62	33.81
**19**	1.456	14	29.74
**20**	1.667	35	34.21
**21**	1.333	61	30.914
**22**	1.183	65	35.19
**23**	1.220	69	32.53
**24**	1.288	94	36.2
**25**	1.252	64	32.85
**26**	1.219	67	31.26

**Table 10:** The coefficients for the X values in the case of the central composite design.

Coefficients for X Values	Y_1_	Y_2_	Y_3_
X_1_	**0.194722**	**−14.3333**	**1.4023**
X_2_	-	**−11.5556**	**1.9642**
X_3_	**0.107556**	**−5.3333**	**0.9361**
X_4_	-	**−2.2778**	**1.34511**
X_1_^2^	-	**-**	0.9498
X_2_^2^	**0.353415**	**−24.8056**	0.9602
X_3_^2^	-	-	1.4298
X_4_^2^	**0.049915**	-	-
X_1_X_2_	**0.131125**	2.625	-
X_1_X_3_	**0.086**	-	**1.1137**
X_1_X_4_	−0.0865	−2.25	1.1445
X_2_X_4_	0.04	-	-
X_3_X_4_	0.046125	-	−0.8927

Note: Significant factors are in bold.

**Table 11:** Direct pelletization process validation at fixed binder amount (3 g) and liquid volume (55 mL).

Experiment No.	Impeller Speed (rpm)	Chopper Speed (rpm)	Aspect Ratio		Yield Percent (%)		Hardness (N)	
			Expected Value	Practical Value	Expected Value	Practical Value	Expected Value	Practical Value
**1**	500	1500	1.204	1.192	87.31	83.36	34.99	34.72
**2**	600	1300	1.16	1.14	84.73	80.02	34	34.02
**3**	700	1200	1.204	1.23	80.83	77.93	33.43	33.55

## Data Availability

Data are available upon request.

## Supplementary Materials

The following supporting information can be downloaded at: https://www.mdpi.com/article/10.3390/pharmaceutics16070848/s1, Figure S1 ANOVA table of aspect ratio results. Figure S2 ANOVA table of yield results. Figure S3 ANOVA table of hardness results. Figure S4 Calibration curves for content uniformity. Figure S5 Calibration curves for dissolution study (HCl). Figure S6 Calibration curves for dissolution study (phosphate buffer).
