# Supplementary material for: Investigation of the Electrokinetic Potential of Granules and Optimization of the Pelletization Method Using the Quality by Design Approach
Source: Pharmaceutics. 2024 Jun 22;16(7):848. doi: 10.3390/pharmaceutics16070848 (PMC11280117; doi:10.3390/pharmaceutics16070848)
Supplement: Supplementary file 1 [file pharmaceutics-16-00848-s001.zip › pharmaceutics-3013579-supplementary.pdf]

Supplementary materials

Factor	ANOVA; Var.:Aspect ratio; R-sqr=.91411; Adj:.8658 (2**(4) central composite, nc=16 ns=8 n0=2 Runs=26 ([No active dataset]) in design space final) 4 factors, 1 Blocks, 26 Runs; MS Residual=.0134704 DV: Aspect ratio				
	SS	df	MS	F	p
(1) Impeller speed(rpm)(L)	0.682501	1	0.682501	50.66677	0.000002
(2) Chopper speed(rpm)(Q)	0.409402	1	0.409402	30.39273	0.000047
(3) Binder amount(g)(Q)	0.008167	1	0.008167	0.60627	0.447559
(4) Liquid amount(ml)(L)	0.208228	1	0.208228	15.45816	0.001192
1L by 2L	0.275100	1	0.275100	20.42258	0.000349
1L by 3L	0.119716	1	0.119716	8.88734	0.008819
1L by 4L	0.118336	1	0.118336	8.78489	0.009142
2L by 3L	0.025600	1	0.025600	1.90046	0.186998
3L by 4L	0.034040	1	0.034040	2.52704	0.131472
Error	0.215526	16	0.013470		
Total SS	2.509457	25			

Figure S1 ANOVA table of aspect ratio results

Factor	ANOVA; Var.:Yield percent; R-sqr=.93101; Adj:.90418 (2**(4) central composite, nc=16 ns=8 n0=2 Runs=26 ([No active dataset]) in design space final) 4 factors, 1 Blocks, 26 Runs; MS Residual=42.43056 DV: Yield percent				
	SS	df	MS	F	p
(1) Impeller speed(rpm)(L)	3698.00	1	3698.000	87.15417	0.000000
(2) Chopper speed(rpm)(L)	2403.56	1	2403.556	56.64681	0.000001
(3) Chopper speed(rpm)(Q)	3407.90	1	3407.902	80.31716	0.000000
(4) Binder amount(g)(L)	93.39	1	93.389	2.20098	0.155222
(5) Liquid amount(ml)(L)	512.00	1	512.000	12.06678	0.002710
1L by 2L	110.25	1	110.250	2.59836	0.124369
1L by 3L	81.00	1	81.000	1.90900	0.183984
Error	763.75	18	42.431		
Total SS	11069.85	25			

Figure S2 ANOVA table of yield results

Hardness response

Factor	ANOVA; Var.:Hardness; R-sqr=.83498; Adj:.72496 (2**(4) central composite, nc=16 ns=8 n0=2 Runs=26 ([No active dataset]) in design space final) 4 factors, 1 Blocks, 26 Runs; MS Residual=2.919908 DV: Hardness				
	SS	df	MS	F	P
(1) Impeller speed(rpm)(L)	35.3977	1	35.39770	12.12288	0.003346
(2) Impeller speed(rpm)(Q)	2.5078	1	2.50781	0.85887	0.368728
(3) Chopper speed(rpm)(L)	69.4470	1	69.44704	23.78398	0.000201
(4) Chopper speed(rpm)(Q)	2.5626	1	2.56258	0.87762	0.363693
(5) Binder amount(g)(L)	32.5678	1	32.56783	11.15372	0.004479
(6) Liquid amount(ml)(L)	15.7735	1	15.77347	5.40204	0.034565
(7) Liquid amount(ml)(Q)	5.6829	1	5.68287	1.94625	0.183306
1L by 3L	20.9581	1	20.95808	7.17765	0.017162
1L by 4L	19.8470	1	19.84702	6.79714	0.019816
3L by 4L	12.7520	1	12.75204	4.36728	0.054082
Error	43.7986	15	2.91991		
Total SS	265.4091	25			

Figure S3 ANOVA table of hardness results

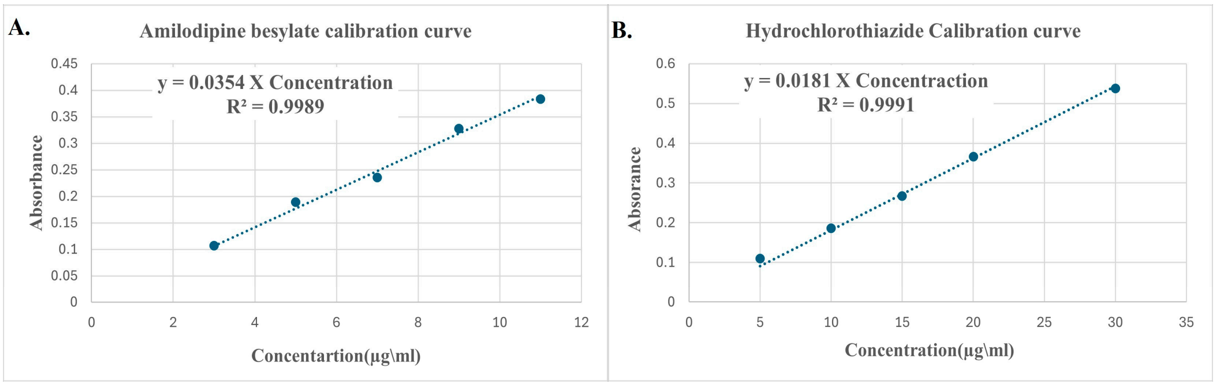

Figure S4 Calibration curves for content uniformity

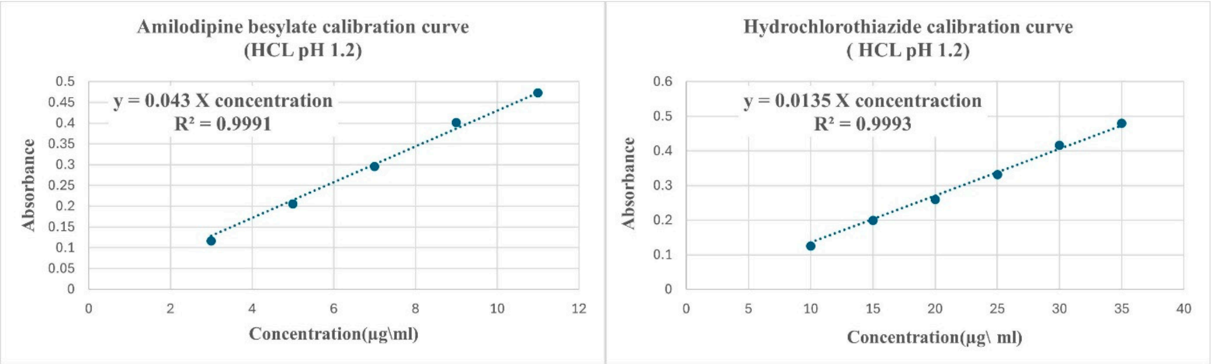

Figure S5 Calibration curves for dissolution study (HCl)

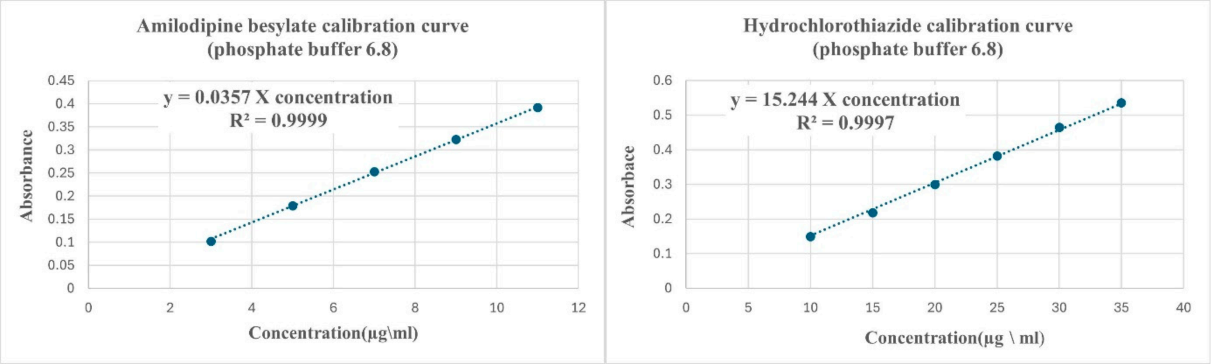

Figure S6 Calibration curves for dissolution study (phosphate buffer)
